# Reduced Chemical Fertilizer Combined With Bio-Organic Fertilizer Affects the Soil Microbial Community and Yield and Quality of Lettuce

**DOI:** 10.3389/fmicb.2022.863325

**Published:** 2022-04-21

**Authors:** Ning Jin, Li Jin, Shuya Wang, Jinwu Li, Fanhong Liu, Zeci Liu, Shilie Luo, Yue Wu, Jian Lyu, Jihua Yu

**Affiliations:** ^1^College of Horticulture, Gansu Agricultural University, Lanzhou, China; ^2^Gansu Provincial Key Laboratory of Arid Land Crop Science, Gansu Agricultural University, Lanzhou, China

**Keywords:** chemical fertilizer, bio-organic fertilizer, high-throughput sequencing, bacterial community, fungal community

## Abstract

Reducing chemical fertilizers in combination with bio-organic fertilizers can limit the use of chemical fertilizers while maintaining soil fertility. However, the effects of combined fertilization on soil chemical properties, microbial community structure, and crop yield and quality are unknown. Using high-throughput sequencing, we conducted field experiments using lettuce plants subjected to five fertilization treatments: chemical fertilizer with conventional fertilization rate (CK), chemical fertilizer reduction by 30% + 6,000 kg ha^–1^ bio-organic fertilizer (T1), chemical fertilizer reduction by 30% + 9,000 kg ha^–1^ bio-organic fertilizer (T2), chemical fertilizer reduction by 40% + 6,000 kg ha^–1^ bio-organic fertilizer (T3), and chemical fertilizer reduction by 40% + 9,000 kg ha^–1^ bio-organic fertilizer (T4). Compared with CK, the T1–T4 had significantly higher soil pH and soil organic matter (SOM) and showed increased richness and diversity of the bacterial community, and decreased richness and diversity of the fungal community. Principal coordinate analysis evidenced that the bacterial and fungal communities of CK and T1–T4 were distinctly separated. The Kruskal-Wallis *H*-test demonstrated that the fungal community was more sensitive than the bacterial community to chemical fertilizer reduction combined with bio-organic fertilizer. Among the soil chemical parameters measured, only TN (total nitrogen) was significantly correlated with bacterial and fungal community composition. The T1 and T2 increased lettuce yield. Moreover, T1–T4 characterized reduced nitrate content and increased levels of soluble sugars and vitamin C in lettuce. Overall, the combined application of reduced chemical fertilizer and bio-organic fertilizer effectively improved soil fertility, microbial community structure, and lettuce yield and quality. These findings have valuable implications for vegetable safety and long-term environmental sustainability.

## Introduction

Fertilization is a globally used management strategy for improving soil quality, and the application of chemical fertilizers is an important determinant of high agricultural production (Yang et al., 2019a). In recent decades, the growth of global crop yields has largely depended on heavy investments in chemical fertilizers (Geng et al., 2019), and farmers manage their farmlands with high fertilization rates to maintain soil productivity. To date, large amounts of chemical fertilizers have been applied to farmlands to obtain higher crop yields (Gruzdeva et al., 2007). However, the amount of chemical fertilizers used is often far greater than that required by crops (Zhu et al., 2019). Excess nutrients in the soil may cause potential damage to the soil, including soil acidification, a decline in soil organic matter (SOM), and a sharp decrease in soil biodiversity (Lv et al., 2020; Wu et al., 2020). Furthermore, it can cause a wide range of environmental problems in marine, freshwater, and terrestrial ecosystems (Tilman et al., 2011). The SOM plays a major role in regulating atmospheric CO_2_ concentrations and maintaining soil fertility and productivity (Lin et al., 2019). Moreover, organic fertilization is the most effective technique for increasing SOM content and improving soil fertility (Liang et al., 2014; Wang et al., 2017b). Numerous studies have shown that organic fertilization reduces soil’s bulk density and increases hydraulic conductivity and soil organic carbon (SOC) content, which forms the basis of soil fertility (Miller et al., 2008; Bandyopadhyay et al., 2010; Victoria et al., 2012). The application of organic fertilizers also increases soil pH and helps alleviate soil acidification (Afreh et al., 2018).

The development of techniques to improve crop yields, the quality of agricultural products, and the sustainability of agricultural production have attracted increasing attention. Belay et al. (2002) reported that long-term inorganic fertilization increased the levels of total organic carbon in soil and significantly increased grain yield in maize. However, the excessive use of chemical fertilizers causes the accumulation of nitrate in vegetable products, leading to a decline in food safety and quality. The negative effects of excessive use of chemical fertilizers can be minimized by reducing the use of chemical fertilizers and combining them with organic fertilizers, as products from organic agricultural systems have generally better nutritional properties (Luthria et al., 2010; Vallverdú-Queralt et al., 2012; Oliveira et al., 2013). Lettuce (*Lactuca sativa* var. *angustana*) is a popular edible vegetable with stems and leaves. China is the largest producer of lettuce, accounting for approximately half of the world’s total lettuce production and cultivation, mainly for stem lettuce products (Nie et al., 2017). The growth cycle of lettuce is relatively short (approximately 90 days). Therefore, although organic fertilizers are more environmentally friendly, nutrient release from organic fertilizers is too slow to support the production of lettuce in a short time (Xiao et al., 2017). The nutrient content of chemical fertilizers is higher than that of organic fertilizers, but it can easily cause soil degradation and environmental pollution. Several studies have found substantive evidence to show that combining chemical and organic fertilizers can improve the quality of a variety of fruits (Wu et al., 2021a). Caris-Veyrat et al. (2004) reported that the levels of carotenoids, polyphenols, and vitamin C were higher in organically cultivated tomatoes than in traditional agricultural tomatoes. In addition, the use of inorganic-organic compound fertilizers not only reduces the use of chemical fertilizers, but also improves the long-term efficiency and sustainability of agroecosystems (Geng et al., 2019).

Soil microorganisms play a crucial role in energy flow and nutrient cycling in soil, and participate in the decomposition of organic matter, degradation of xenobiotics, soil carbon sequestration, and prevention of crop diseases (Morris and Blackwood, 2015; Tao et al., 2015). Bacteria and fungi are indicator species of the soil environment (Hermans et al., 2017; Wolińska et al., 2021), and are conducive to the improvement of the soil structure and plant growth. Fungi can grow symbiotically with crops to form mycorrhizae, which are of major significance for promoting plant growth and maintaining agroecosystem stability (Brito et al., 2012). Bacteria and fungi also have a cooperative relationship in soil environments that lack nutrients; that is, fungal hyphae are beneficial for improving and balancing nutrients in the soil, thereby promoting the growth and metabolism of bacteria (Strickland and Rousk, 2010). Soil microbial biomass and diversity are potentially valuable indicators of soil quality and are sensitive to changes in soil nutrients, pH, and organic matter content (Zhong et al., 2010). Microorganisms can quickly respond to changes in environmental factors by modifying microbial diversity and community composition (Krashevska et al., 2015; Wolińska et al., 2017). Soil microbial communities are greatly affected by anthropogenic activities such as agricultural intensification and fertilization. Moreover, nutrient sources and application rates determine nutrient availability in soil; therefore, the application of different nutrient sources may cause significant shifts in the predominant microbial taxa (Hartmann et al., 2015; Sun et al., 2015). Previous studies have reported changes in soil microbial communities after fertilization. Based on 27 years of experiments, Lin et al. (2019) reported that organic fertilizers had a strong impact on soil microbial community structure and led to an increase in the abundance of Bacillales, Gaiellales, and Pezizales. Regardless of whether chemical fertilizers are used, organic fertilization has a positive effect on bacterial and fungal diversity (Kamaa et al., 2011). The combined application of organic and inorganic fertilizers can accelerate microbial growth, change the soil microbial community structure, and improve enzyme activity (Lazcano et al., 2013). Therefore, the use of organic fertilizers to maintain the stability of underground ecosystem function has attracted increased attention.

Previous studies have primarily focused on long-term chemical or organic fertilization alone, and few studies have investigated the use of bio-organic fertilizers, combined with reduced chemical fertilizers, especially in lettuce. As a result, the variation in yield, quality, and soil bacterial and fungal community diversity in organic-inorganic compound fertilization systems remains unclear. As discussed above, excessive chemical fertilization has negative effects on the environment, whereas organic fertilization has beneficial effects on crop’s growth and quality. Exploring how the soil’s bacterial and fungal communities respond to the agricultural fertilization system would be conducive to the development of sustainable agriculture. Our objectives were as follows: (i) to evaluate how different degrees of reduction of chemical fertilizers combined with bio-organic fertilizers affected crop yield and quality, soil nutrients, soil microbial (bacterial and fungal) diversity and composition, and soil microbial community structure; (ii) to assess the relationship between soil chemical properties and microbial communities. The results of this study provide scientific support for the establishment of an environmentally friendly fertilization technology and effectively promote the sustainable development of agriculture.

## Materials and Methods

### Field Site and Experiment Design

The study was conducted in Tianzhu, Gansu, China (37° 09′ N, 102° 99′ E), in May 2019 and 2020. The altitude of this region is approximately 2,630 m, the annual average temperature is −2°C, and the average annual precipitation is 400–450 mm. At the study location, the soil type was loam, the terrain was flat, and the soil fertility was uniformly moderate. Lettuce seeds (cv. “Yong’an lettuce—-Zilong lettuce”) were sown at Fuzhou Kexiang Seed Industry Co., Ltd., and thinned thereafter. The conventional fertilizers used in this study were urea (N ≥ 46%), calcium superphosphate (P_2_O_5_ ≥ 16%), and potassium sulfate (K_2_O ≥ 52%). The bio-organic fertilizer used in the experiment (organic matter content ≥ 40%, *Bacillus subtilis*, and *Pseudomonas stutzeri* with effective viable count ≥ 20 million CFU/g) was purchased from Gansu Lvneng Agricultural Technology Co., Ltd. The calcium superphosphate and bio-organic fertilizers are both applied as basal fertilizers. In this study, 30% of the urea and potassium sulfate were used as basal fertilizers. Moreover, 70% of the urea and potassium sulfate were top-dressed twice; of this, 30% was applied in the seedling stage and 40% in the rhizome expansion stage.

Five treatments were used in this experiment: (i) chemical fertilizer with conventional fertilization, as used by local farmers (CK; NPK dosage: 965.2 kg ha^–1^: 314.3 kg N ha^–1^, 478.5 kg P ha^–1^, and 172.4 kg K ha^–1^); (ii) chemical fertilizer reduction by 30% + 6,000 kg ha^–1^ bio-organic fertilizer (T1; NPK dosage: 675.6 kg ha^–1^: 293.9 kg N ha^–1^, 117.2 kg P ha^–1^, and 264.5 kg K ha^–1^); (iii) chemical fertilizer reduction by 30% + 9,000 kg ha^–1^ bio-organic fertilizer (T2; NPK dosage: 675.6 kg ha^–1^: 293.9 kg N ha^–1^, 117.2 kg P ha^–1^, and 264.5 kg K ha^–1^); (iv) chemical fertilizer reduction by 40% + 6,000 kg ha^–1^ bio-organic fertilizer (T3; NPK dosage: 579.1 kg ha^–1^: 251.9 kg N ha^–1^, 100.5 kg P ha^–1^, and 226.7 kg K ha^–1^); and (v) chemical fertilizer reduction by 40% + 9,000 kg ha^–1^ bio-organic fertilizer (T4; 579.1 kg ha^–1^: 251.9 kg N ha^–1^, 100.5 kg P ha^–1^, and 226.7 kg K ha^–1^). Each treatment included three replicates, and the area of each experimental plot was 80 m^2^. We used the ridge, double row, and semi-film cultivation modes for the experiments. The cultivation density was 57,750 plants ha^–1^, the ridge width was 50 cm, the furrow width was 40 cm, and the plant spacing was 30 cm. We ensured that all field management practices (other than fertilization) were consistent across treatments.

### Soil Sampling and Analysis of Soil Chemical Properties

After lettuce harvesting in August 2020, soil samples were collected from the plow layer (0–20 cm) of each replicate plot for each treatment using a stainless-steel auger. Each soil sample was a composite of five randomly collected soil cores that were pooled in a sterile plastic bag and transported to the laboratory on ice. The soil samples were sieved using a 2-mm mesh sieve and divided into two subsamples before thorough homogenization. One part was air-dried to determine the physical and chemical properties of the soil, and the other part was stored at −80°C for DNA extraction.

The soil pH and electrical conductivity (EC) were measured after shaking the soil-water suspension (1:5 wt/vol) for 30 min. The pH of the filtrate was determined using a glass electrode (PHS-3E, Shanghai Jingke, China), and the EC was measured by inserting a conductivity meter (DSJ-308A, Shanghai Jingke) into the filtrate. The SOM content was measured using a titration method after oxidation with K_2_Cr_2_O_7_. The soil’s total nitrogen (TN), total phosphorus (TP), and total potassium (TK) were determined using the H_2_SO_4_-H_2_O_2_ wet digestion method. The TN was measured using the Kjeldahl method using a fully automatic Kjeldahl K1100F apparatus (Jinan Hanon Instruments Company, Jinan, China). The TP was measured using the Mo-Sb colorimetric method and analyzed using a UV-1780 spectrophotometer [Shimadzu Instruments (Suzhou) Co., Ltd., Suzhou, China]. The TK was analyzed using a ZEEnit 700P atomic absorption spectrometer (Analytik Jena, Germany). All samples were tested in triplicates.

### Soil DNA Extraction and PCR Amplification

The genomic DNA of the soil microbial community was extracted from 0.5 g soil samples using the E.Z.N.A.^®^ soil DNA Kit (Omega Bio-tek, GA, United States) according to the manufacturer’s instructions. The DNA extract was analyzed on a 1% agarose gel, and DNA concentration and purity were determined using a NanoDrop ND-2000 UV-VIS spectrophotometer (Thermo Scientific, Wilmington, DE, United States). The hypervariable V3–V4 region of the bacterial 16S rRNA gene was amplified with the primer pairs 338F (5′-ACTCCTACGGGAGGCAGCAG-3′) and 806R (5′-GGACTACHVGGGTWTCTA AT-3′) (Chen et al., 2016) using an ABI GeneAmp 9700 PCR thermocycler (Applied Biosystems, CA, United States). The internal transcribed spacer (ITS) region of fungi was amplified using the primers ITS1F (5′-CTTGGTCATTTAGAGGAAGTAA-3′) and ITS2R (5′-GCTGCGTTCTTCATCGATGC-3′) (Blaalid et al., 2013) on the same thermal cycler. The PCR was conducted using 20 μl reaction mixtures containing the template DNA (10 ng), 4 μL of 5 × TransStart FastPfu buffer (TransGen Biotech, Beijing, China), 2 μL of 2.5 mM dNTPs, 0.8 μL each of the forward and reverse primers (5 μm), 0.4 μl of TransStart FastPfu DNA Polymerase, and ddH_2_O. The PCR conditions were as follows: initial denaturation at 95°C for 3 min, 27 cycles of denaturation at 95°C for 30 s, annealing at 55°C for 30 s, and extension at 72°C for 45 s, a single extension at 72°C for 10 min, and storage at 4°C. The PCRs were performed in triplicate. The PCR product was extracted from a 2% agarose gel, purified using the AxyPrep DNA gel extraction kit (Axygen Biosciences, CA, United States) according to the manufacturer’s instructions, and quantified using a Quantus Fluorometer (Promega, WA, United States).

### Illumina Miseq Sequencing and Sequence Processing

Purified amplicons were pooled in equimolar amounts and paired-end sequenced (2 × 300 bp) on an Illumina MiSeq PE300 platform (Illumina, CA, United States) by Shanghai Majorbio Bio-pharm Technology Co., Ltd. (Shanghai, China). The raw sequences were analyzed using the Fastp software (version 0.19.6)^1^ (Chen et al., 2018) for quality control. The paired-end reads were merged using the FLASH software (version 1.2.11)^2^ (Magoč and Salzberg, 2011). The reads were dereplicated, sorted, and clustered into operational taxonomic units (OTUs) at the default 97% similarity using UPARSE (version 7.0.1090)^3^ (Edgar, 2013), and dechimerized against the UCHIME reference dataset (Edgar et al., 2011). Taxonomic labels were assigned to the OTUs using the Silva 16S bacterial database and the UNITE fungal database (version 7.2) (Nilsson et al., 2019) using the RDP classifier (version 2.11)^4^ with a minimum confidence threshold of 0.7.

### Determination of the Yield and Quality of Lettuce

In August 2019 and 2020, the lettuce crops were harvested after they had reached commercial standards. The yields of all plots were determined. The nitrate content was determined using the salicylic acid-sulfuric acid method (Cataldo et al., 1975). The 2,6-dichloroindophenol staining method was used to determine the vitamin C content (Arya et al., 2000), and the Coomassie Brilliant Blue method was used to estimate the levels of soluble protein (Sedmak and Grossberg, 1977). The soluble sugar content was measured using the anthrone-sulfuric acid assay (Wang et al., 2020).

### Statistical Analysis

Microsoft Excel 2013 was used to calculate the mean and standard error (SE) of the crop yield and quality of lettuce and the chemical properties of soil in different treatment groups. One-way analysis of variance (ANOVA) was applied to evaluate the effects of different treatments on the lettuce yield and quality and soil chemical properties. Duncan’s multiple range test (significance level: *p* < 0.05) was used to compare the differences in mean values among different fertilization treatments. The SPSS software (version 23.0, IBM Corp., NY, United States) was used for one-way ANOVA.

Alpha diversity indices (coverage, ACE, Chao1, Shannon index, and Simpson index) at the OTU level were calculated using the Mothur software (version 1.30.2). For the analysis of beta diversity, we used unweighted UniFrac distances to generate principal coordinate analysis (PCoA) maps to assess the differences between the bacterial community members of soil samples with different fertilization treatments (Han et al., 2021). In addition, we used PCoA based on Bray–Curtis distances to evaluate differences between the fungal community members of soil samples with different fertilization treatments. Redundancy analysis (RDA) was applied to analyze the correlation between microbial community composition and soil chemical properties (Yang et al., 2019c). The Mantel test based on Bray–Curtis distances was used to determine the relationship between microbial community composition and soil properties at the OTU level. The PCoA, heatmap, and RDA were computed using the *vegan* package in R (Oksanen et al., 2015).

## Results

### Chemical Properties of Soils With Different Fertilization Treatments

Table 1 lists the chemical properties of soils with different fertilization treatments. Compared with the CK treatment, the four treatments (T1, T2, T3, and T4) with reduced chemical fertilizer combined with bio-organic fertilizer significantly increased the soil pH (Table 1). The SOM values of the T1, T2, T3, and T4 treatment groups were significantly higher compared to those in the CK treatment group by 61.94, 59.18, 65.60, and 71.12%, respectively. The EC in the T1, T2, T3, and T4 treatment groups was significantly lower than that in the CK group; however, the EC of T1 and T2 was significantly higher than that of T3 and T4. The T2 treatment group had the highest TN and TK levels, which were significantly higher (by 35.71 and 16.69%, respectively) than that in the CK group. There was no significant difference in TP among the T2, T3, T4, and CK treatments.

**TABLE 1:** Soil chemical properties with different fertilization treatments.

Treatments	pH	EC (μ Scm^–1^)	SOM (g kg^–1^)	TN (g kg^–1^)	TP (g kg^–1^)	TK (g kg^–1^)
CK	6.33 ± 0.10*^c^*	485.33 ± 5.90*^a^*	27.56 ± 0.91*^c^*	0.42 ± 0.02*^b^*	0.29 ± 0.01*^a^*	6.77 ± 0.25*^c^*
T1	6.72 ± 0.05*^b^*	408.33 ± 6.44*^b^*	44.63 ± 0.33*^ab^*	0.56 ± 0.02*^a^*	0.22 ± 0.02*^b^*	7.23 ± 0.16*^bc^*
T2	7.12 ± 0.02*^a^*	418.00 ± 8.62*^b^*	43.87 ± 0.55*^b^*	0.57 ± 0.02*^a^*	0.28 ± 0.02*^a^*	7.90 ± 0.10*^a^*
T3	7.04 ± 0.08*^a^*	258.67 ± 3.48*^d^*	45.64 ± 1.01*^ab^*	0.41 ± 0.01*^b^*	0.28 ± 0.01*^a^*	7.73 ± 0.17*^ab^*
T4	7.05 ± 0.04*^a^*	306.00 ± 11.53*^c^*	47.16 ± 1.01*^a^*	0.49 ± 0.06*^ab^*	0.27 ± 0.01*^a^*	7.92 ± 0.10*^a^*

*Values indicate mean ± SE (n = 3). Different superscript letters in the columns represent significant differences among fertilizer treatments according to one-way ANOVA (Duncan’s test, p < 0.05). EC, electrical conductivity; SOM, soil organic matter; TN:, total nitrogen; TP, total phosphorus; TK, total potassium; CK, chemical fertilizer with conventional fertilization rate; T1, chemical fertilizer reduction by 30% + 6,000 kg ha^–1^ bio-organic fertilizer; T2, chemical fertilizer reduction by 30% + 9,000 kg ha^–1^ bio-organic fertilizer; T3, chemical fertilizer reduction by 40% + 6,000 kg ha^–1^ bio-organic fertilizer; T4, chemical fertilizer reduction by 40% + 9,000 kg ha^–1^ bio-organic fertilizer.*

### Alpha Diversity of Bacterial and Fungal Communities in Soils With Different Fertilization Treatments

Coverage refers to the coverage of each sample library. The higher the value, the higher the probability that the sequence in the sample will be detected. This index reflects whether the sequencing results represent the actual microorganisms in the sample. The coverage indices for both bacteria and fungi exceeded 0.96 (Table 2), indicating that the sequencing results reflected the actual microorganisms in the soil samples. The higher the values of community richness indices (Chao1 and ACE), the higher the community richness. In terms of the bacterial community, the Chao1 and ACE indices in the T1–T4 treatment groups were higher (2,950.93–3,058.16 and 2,942.68–3,060.67, respectively) than those in the CK treatment group (2,859.13 and 2,864.02, respectively). In particular, the Chao1 and ACE indices of the T3 treatment group were significantly higher than those of the CK group, although there was no significant difference between the other three treatment groups and the CK treatment group (Table 3, *p* < 0.05). For the fungal community, the Chao1 and ACE indices of the T1–T4 treatment groups were lower (582.66–690.62 and 585.28–695.51, respectively) than those in the CK treatment group (723.01 and 708.37, respectively). In particular, the Chao1 and ACE indices of the T2 treatment group were significantly lower than those of the CK treatment group, and there was no significant difference between the other three treatment groups and the CK treatment group (Table 3, *p* < 0.05). We also used the Shannon and Simpson diversity indices to assess the diversity of each sample. The higher the Shannon index, the higher the microbial diversity of the sample, and the higher the Simpson index, the lower the microbial diversity of the sample. The Shannon indices of bacteria were higher in the T2 and T3 treatment groups (6.393 and 6.408, respectively) than in the CK treatment group (6.388), but the difference was not significant (Table 2). The Simpson index of bacteria was the lowest in the T2 treatment group. The Shannon indices of fungi were significantly lower in the T1–T4 treatment groups than in the CK treatment group (*p* < 0.05), whereas the Simpson index showed the opposite trend.

**TABLE 2:** Alpha diversity indices of soil bacteria and fungi in different fertilization treatments.

	Treatments	Coverage	Chao1	ACE	Shannon	Simpson
Bacteria	CK	0.9632 ± 0.001*^b^*	2859.13 ± 46.73*^b^*	2864.02 ± 32.10*^b^*	6.388 ± 0.032*^a^*	0.0065 ± 0.0003*^ab^*
	T1	0.9717 ± 0.0019*^a^*	2933.55 ± 18.16*^ab^*	2966.92 ± 11.72*^ab^*	6.344 ± 0.015*^a^*	0.0069 ± 0.0003a
	T2	0.9712 ± 0.0014*^a^*	2950.93 ± 28.44*^ab^*	2971.51 ± 29.20*^ab^*	6.393 ± 0.025*^a^*	0.0059 ± 0.0001*^b^*
	T3	0.9660 ± 0.0013*^b^*	3058.16 ± 81.43*^a^*	3060.67 ± 58.18*^a^*	6.408 ± 0.020*^a^*	0.0065 ± 0.0002*^ab^*
	T4	0.9648 ± 0.0016*^b^*	2961.87 ± 43.19*^ab^*	2942.68 ± 27.06*^ab^*	6.388 ± 0.015*^a^*	0.0062 ± 0.0003*^ab^*
Fungi	CK	0.9977 ± 0.0002*^a^*	723.01 ± 15.31*^a^*	708.37 ± 11.64*^a^*	4.009 ± 0.011*^a^*	0.0428 ± 0.0011*^b^*
	T1	0.9980 ± 0.0002*^a^*	658.25 ± 20.54*^a^*	658.3 ± 22.37*^ab^*	3.928 ± 0.018*^b^*	0.0457 ± 0.0012*^b^*
	T2	0.9982 ± 0.0001*^a^*	582.66 ± 13.21*^b^*	585.28 ± 13.33*^c^*	3.764 ± 0.010*^c^*	0.0542 ± 0.0004*^a^*
	T3	0.9978 ± 0.0001*^a^*	690.62 ± 8.01*^a^*	695.51 ± 5.11*^ab^*	3.753 ± 0.016*^c^*	0.0574 ± 0.0004*^a^*
	T4	0.9977 ± 0.0004*^a^*	660.17 ± 33.35*^a^*	647.00 ± 23.91*^b^*	3.725 ± 0.033*^c^*	0.0587 ± 0.0033*^a^*

*Values indicate mean ± SE (n = 3). Different superscript letters in the columns represent significant differences among fertilizer treatments according to one-way ANOVA (Duncan’s test, p < 0.05). The abbreviations CK, T1, T2, T3, and T4 are as defined in the footnote to Table 1.*

**TABLE 3:** Mantel test results for correlations between soil chemical properties and bacterial and fungal community composition at the OTU level.

Parameters	Bacterial composition		Fungal composition	
	*r*	*p*	*r*	*p*
pH	0.360	0.015*	0.173	0.154
EC	0.154	0.180	0.020	0.864
SOM	0.351	0.037*	0.040	0.789
TN	0.226	0.016*	0.221	0.029*
TP	0.092	0.557	0.134	0.288
TK	0.407	0.006**	0.014	0.902

*p < 0.05; **p < 0.01. CK, T1, T2, T3, and T4 are as defined in the footnote to Table 1.

### Beta Diversity of Bacterial and Fungal Communities in Soils With Different Fertilization Treatments

**FIGURE 1:**
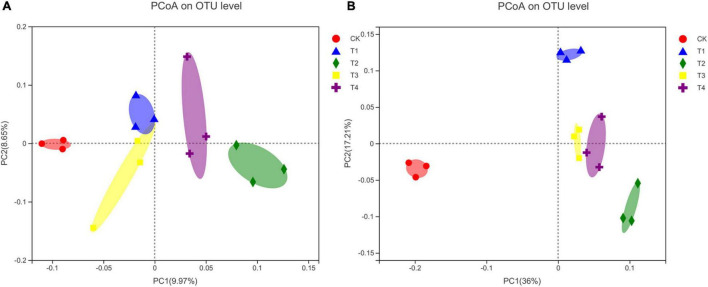
Principal coordinate analysis (PCoA) plots of bacterial **(A)** and fungal **(B)** community composition at the OTU level. CK, chemical fertilizer with conventional fertilization rate; T1, chemical fertilizer reduction by 30% + 6,000 kg ha^–1^ bio-organic fertilizer; T2, chemical fertilizer reduction by 30% + 9,000 kg ha^–1^ bio-organic fertilizer; T3, chemical fertilizer reduction by 40% + 6,000 kg ha^–1^ bio-organic fertilizer; T4, chemical fertilizer reduction by 40% + 9,000 kg ha^–1^ bio-organic fertilizer.

The PCoA analysis showed that the community composition of soil bacteria and fungi was altered by the reduction of chemical fertilizer combined with bio-organic fertilizer (Figure 1). For the bacterial community structure, the first and second principal coordinates explained 9.97 and 8.65% of the differences among the five treatments, respectively (Figure 1A). Moreover, the bacterial communities of the T1–T4 treatment groups were considerably separated from those of the CK treatment group (Figure 1A). The bacterial community structures of the T1 and T3 treatment groups appeared to be similar. For the fungal community structure, the first and second principal coordinates explained 36 and 17.21% of the variance, respectively (Figure 1B). The fungal communities of the T1–T4 treatment groups were clearly distinguished from those of the CK treatment group along the first principal component axis. Moreover, the fungal community structure was similar between the T3 and T4 treatment groups.

### Composition and Relative Abundance of Bacterial and Fungal Communities in Soils With Different Fertilization Treatments

**FIGURE 2:**
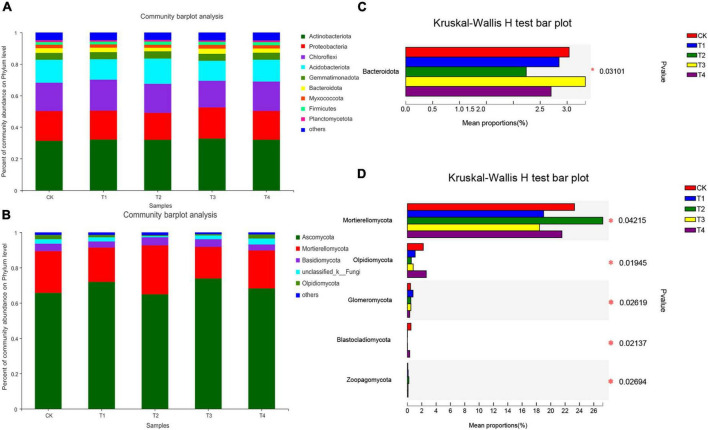
Relative abundances of bacterial **(A)** and fungal **(B)** taxa at the phylum level. The relative abundances of bacteria **(C)** and fungi **(D)** were tested by the Kruskal–Wallis *H*-test. “Other” includes phyla with <1% average relative abundance. Values for individual treatments are the means of three replicate soil samples. The false discovery rate was used to control for multiple tests, and the Scheffe test was used to calculate the 95% confidence intervals. CK, T1, T2, T3, and T4 are as defined in the Figure 1 legend.

The phylum composition of the bacteria in the five treatment groups is shown in Figure 2A. The top five bacterial phyla were Actinobacteria (31.25–32.68%), Proteobacteria (17.22–19.88%), Chloroflexi (16.86–19.54%), Acidobacteria (12.65–16.01%), and Gemmatimonadota (4.31–4.61%). These five bacterial phyla accounted for more than 85% of the total sequence reads. Compared to the CK group, the relative abundance of Actinobacteria was higher in the T1, T2, T3, and T4 treatment groups. The relative abundance of Gemmatimonadota in the CK treatment was lower than those in the T1–T4 treatment groups. There was no significant difference in the relative abundance of other phyla among all treatments, except for Bacteroidetes (*p* < 0.05). The relative abundance of Bacteroidetes was significantly lower in the T1, T2, and T4 groups than in the CK treatment group (Figure 2C, *p* < 0.05).

The dominant fungal phyla in all treatment groups were Ascomycota, Mortierellomycota, and Basidiomycota, which accounted for 65.10–72.87%, 18.39–27.28%, and 3.37–4.70% of all sequence reads, respectively (Figure 2B). Overall, these three fungal phyla accounted for more than 90% of the high-quality sequences. Compared with the CK treatment group, the T2 treatment decreased the relative abundance of Ascomycota and increased that of Basidiomycota. Furthermore, the Kruskal–Wallis *H*-test showed that the relative abundances of Mortierellomycota, Olpidiomycota, Glomeromycota, Blastocladiomycota, and Zoopagomycota were significantly different among the five treatment groups (Figure 2D, *p* < 0.05).

### Relationship Between Microbial Community Structure and Soil Chemical Properties

**FIGURE 3:**
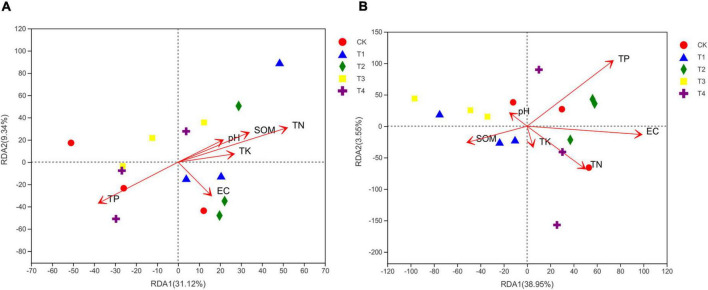
Redundancy analysis (RDA) of bacterial **(A)** and fungal **(B)** communities with soil chemical properties. EC, electrical conductivity; SOM, soil organic matter; TN, total nitrogen; TP, total phosphorus; TK, total potassium. CK, T1, T2, T3, and T4 are as defined in the Figure 1 legend.

The RDA analysis in Figure 3 shows the relationship between soil chemical properties and microbial community composition. The first two axes of the RDA analysis explained 40.46% of the total variance in the soil bacterial community (axis 1: 31.12%; axis 2: 9.34%) (Figure 3A). The TN showed the highest correlation with the soil bacterial community structure, followed by TP, SOM, TK, pH, and EC. The first two axes of the RDA analysis explained 42.50% of the total variance in the soil fungal community (axis 1: 38.95%; axis 2: 3.55%) (Figure 3B). Soil chemical properties influenced the soil fungal community composition in the following order: EC > TP > TN > SOM > pH > TK. The results of a Mantel test (Table 3) revealed that TN, SOM, pH, and TK were significantly (*p* < 0.05) or extremely significantly (*p* < 0.01) positively correlated with bacterial community composition, and that TN was significantly positively correlated with fungal community composition (*p* < 0.01).

### Lettuce Yield and Quality

**FIGURE 4:**
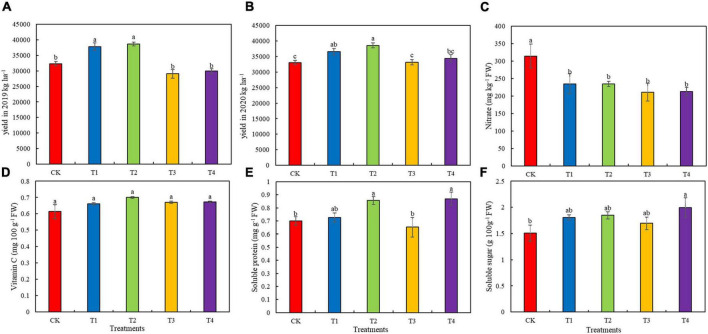
Yield and quality parameters of lettuce grown in soils with different fertilization treatments. **(A)** Yield in 2019. **(B)** Yield in 2020. **(C)** Nitrate content of lettuce. **(D)** Vitamin C content of lettuce. **(E)** Soluble protein content of lettuce. **(F)** Soluble sugar content of lettuce. Values presented are the means ± SE (*n* ≥ 3). Vertical bars indicate the SE of the means. Different lowercase letters indicate significant differences among treatments at the same timepoint (Duncan’ s test, *p* < 0.05). CK, T1, T2, T3, and T4 are as defined in the Figure 1 legend.

Among the five fertilization treatments, the T2 treatment group had the highest yield (Figures 4A,B). The 2-year yields of the T1 and T2 treatment groups were significantly higher than that of the CK group (*p* < 0.05). Compared with the 2-year yield of the CK treatment group, the yield of the T2 treatment group increased by 19.43 and 16.63%, respectively. The nitrate content of lettuce was significantly lower in the T1–T4 treatment groups (by 25.25, 25.46, 32.88, and 32.44%, respectively) than in the CK group (Figure 4B, *p* < 0.05). However, there was no significant difference in vitamin C content among the five fertilization treatments (Figure 4C, *p* < 0.05). The soluble protein content of the T2 and T4 treatment groups were significantly higher (by 22.49 and 24.30%, respectively) compared to that in the CK group (Figure 4D, *p* < 0.05). The soluble sugar content of lettuce was higher in the T1–T4 treatment groups than in the CK group (Figure 4E). Compared to the soluble sugar content of the CK group, that of the T2 treatment increased significantly by 32.19%, and those of T1, T2, and T3 groups increased by 19.62, 22.52, and 12.29%, respectively (Figure 4F, *p* < 0.05).

## Discussion

### Effects of the Reduction of Chemical Fertilizer Combined With Bio-Organic Fertilizer Treatment on Soil Chemical Properties and Microbial Diversity

The results of the present study demonstrated that the soil chemical properties changed significantly after the application of chemical fertilizer combined with bio-organic fertilizer. Specifically, the pH and SOM of the four treatment groups (reduced chemical fertilizer combined with bio-organic fertilizer) were significantly improved compared with the values in the control (CK) group (Table 1, *p* < 0.05). This was consistent with the results of previous studies showing that the application of organic fertilizer improved the soil chemical properties in rice (Wang et al., 2014). The significant increase in soil pH may be ascribed to the mitigation of soil acidification through the decomposition of organic matter (Xiaojing et al., 2018). Organic fertilizers are an important source of SOM, and SOM with a higher C content is important for maintaining soil fertility and improving soil properties (Yang et al., 2019b; Zhu et al., 2019). Pinggera et al. (2015) found that adding an appropriate amount of nitrogen facilitates the microbial decomposition of organic matter and adjusts the C/N ratio to levels more suitable for the growth of soil microorganisms. This accelerates decomposition and the release of organic nutrients by microorganisms and increases the content of SOM, which may explain the increase in SOM in the T1–T4 treatment groups in this study. In addition, the TN content was increased by a 30% reduction in chemical fertilizers combined with 6,000 or 9,000 kg ha^–1^ bio-organic fertilizer (Table 1), which is consistent with the results of Yang et al. (2019b) and Wu et al. (2021b).

Previous studies on arable soil have shown that conventional fertilization with chemical fertilizers reduced bacterial richness and increased fungal richness (as measured using the Chao1 and ACE indices) (Shen et al., 2010; Zhong et al., 2010). In this study, the group with conventional chemical fertilization had the lowest Chao1 and ACE indices for bacteria and the highest indices for fungi (Table 2); thus, these results were consistent with those of the aforementioned studies. Ai et al. (2012) also reported that the application of mineral nitrogen fertilizers generally promotes the growth of fungi. In contrast, we found that the Chao1 and ACE indices were significantly lower in the T2 treatment group (30% fertilizer reduction + 9,000 kg ha^–1^ bio-organic fertilizer) than in the T3 and T4 treatment groups (40% fertilizer reduction + 6,000 or 9,000 kg ha^–1^ bio-organic fertilizer, *p* < 0.05). This may be because among the T1–T4 treatment groups, the T2 group had the highest pH, and studies have shown that within a pH range of 4–8.3, lower pH promotes the growth of fungi (Rousk et al., 2009). There was no significant change in the Shannon diversity index of bacteria in the treatment groups (*p* < 0.05), indicating that the bacteria showed adaptability to the soil under different fertilization systems. Luo et al. (2015) discovered that compared to inorganic fertilizers, organic fertilizers are more conducive to increasing fungal diversity. However, in this study, the Shannon diversity index of fungi was reduced by a treatment with reduced chemical fertilizers combined with bio-organic fertilizer, and the Simpson index showed the opposite trend. Studies using culture-dependent methods have shown that long-term organic fertilization—especially with manure—can reduce fungal diversity (He et al., 2008). These contrasting results are likely due to differences in experimental methods and soil types. Moreover, the PCoA analysis revealed that the bacterial and fungal community compositions were clearly separated between the control and treatment groups. Similar results have also been reported by Yu et al. (2013) and Ding et al. (2017).

### Effects of Reduced Chemical Fertilizer Combined With Bio-Organic Fertilizer on Soil Microbial Community Composition

Inorganic or organic fertilization leads to the enrichment of specific bacteria or fungi that can effectively utilize these nutrients, thereby altering the composition of the microbial community. Elucidating soil microbial taxa at the phylum level can reveal the ecological consistency of a microbial community (Guo et al., 2018). In this study, high-throughput sequencing showed that the dominant bacterial phyla were Actinobacteria, Proteobacteria, Chloroflexi, Acidobacteria, and Gemmatimonadota (Figure 2A), which is consistent with previous studies on agricultural soils (Chen et al., 2016; Guo et al., 2018; Wu et al., 2021b). Actinobacteria are eutrophic bacteria that are considered an indicator taxon and play a key role in the soil carbon and nitrogen cycle (Zhu et al., 2019). The present study showed that the relative abundance of Actinobacteria was increased in the four treatment groups (T1–4). Wang et al. (2017a) reported that the relative abundance of Bacteroidetes increases after 10 years of inorganic fertilization. Similarly, in this study, the relative abundance of Bacteroidetes was significantly higher in the control group (conventional chemical fertilization) than in the combined fertilization groups (reduced chemical fertilizer and bio-organic fertilizer). Consistent with previous studies, Ascomycota was the most abundant fungal phylum in all fertilization treatments (Chen et al., 2016; Bei et al., 2018). Ascomycota and Basidiomycota are typical saprophytes that are environmentally friendly, decompose organic matter (cellulose, lignin, and pectin), and play an important role in degrading litter with high lignin content in the soil (Yelle et al., 2008; Guo et al., 2018; Liu et al., 2019). Compared with the CK treatment group, the T2 group (30% reduction of chemical fertilizer combined with 6,000 kg ha^–1^ bio-organic fertilizer) had a lower relative abundance of Ascomycota and increased relative abundance of Basidiomycota (Figure 2B). This may be due to a competitive relationship between the Ascomycota and Basidiomycota microbes that results in a negative correlation between the relative abundances of the two phyla (Ye et al., 2020a). Several studies have shown that compared with bacteria, fungi are more sensitive to invasive plants (Xiao et al., 2014), salt stress (Chowdhury et al., 2011), soil moisture (Kaisermann et al., 2015), fertilizer compost (Shen et al., 2019) and other factors. Yang et al. (2020b) demonstrated that compared to soil bacteria, soil fungi are more sensitive to the application of organic fertilizer (pig manure). Furthermore, the Kruskal–Wallis *H*- test also showed that the only bacterial phylum with significant differences among the five treatments was Bacteroidetes, whereas the other fungal phyla—including Mortierellomycota, Olpidiomycota, Glomeromycota, Blastocladiomycota, and Zoopagomycota—were significantly different between groups (Figure 2, *p* < 0.05).

### Relationship Between the Microbial Community and Soil Environment Factors

TN is significantly associated with ammonia-oxidizing bacterial communities and is one of the main factors promoting changes in the soil microbial community (Qu et al., 2016; Yang et al., 2017). Actinobacteria is a dominant taxon in the bacterial community and is positively correlated with the soil nitrogen pool; therefore, TN has been proven to drive changes in the soil bacterial community (Nemergut et al., 2010). In this study, TN was found to be the main factor driving changes in the bacterial community (Figure 3A). pH is also considered the most significant factor determining the composition of the soil bacterial community (Zhao et al., 2015; Kim et al., 2016), and the results of the Mantel test showed that TN and pH were significantly correlated with soil bacterial community composition (Table 3, *p* < 0.05). Fungal community composition is affected by soil nutrient content (i.e., TN and TP) (Paungfoo-Lonhienne et al., 2015; He et al., 2016). Accordingly, the main driving factors of the fungal community were EC, TP, and TN in this study (Figure 3B). The results of the Mantel test showed that TN was significantly positively correlated with the fungal community composition (Table 3). Soil nutrients may indirectly affect fungal community composition by regulating changes in the aboveground portions of plants (Haiyan et al., 2016). Previous studies have demonstrated that SOM is the primary driver factor regulating soil fungal community composition (Liu et al., 2015). This is inconsistent with the results of this experiment and suggests that the mineralization of SOM by organic fertilizers is a long-term process. This makes it difficult to assess the full effect of their nutritional potential on the soil microbial community in a short-term study period (Zhang et al., 2021). Thus, the short-term effects of organic fertilizers on soil microbial communities appear to be more modest than those of chemical nitrogen fertilizers.

### Effects of Reduced Chemical Fertilizer Combined With Bio-Organic Fertilizer on Lettuce Yield and Quality

Excessive chemical fertilization is related to several problems, including high levels of N–P–K in vegetable production and the safety of vegetable products in the market. Bio-organic fertilizers are a potential alternative to mineral fertilizers in crop production that can address these urgent problems (Naeem et al., 2006; Flores-Félix et al., 2013). The application of bio-organic fertilizers to improve plant nutrition can help reduce mineral fertilization and is becoming a popular strategy for sustainable agriculture. Bio-organic fertilizers are a new type of organic fertilizer that contains various beneficial microbial communities. These unique microbial communities can activate the soil, improve its physical and chemical properties, and increase soil microbial diversity (Bandyopadhyay et al., 2010; Xue et al., 2010). However, reducing the use of chemical fertilizers in agriculture without causing loss of productivity is a feasible—but difficult—challenge. Bio-organic fertilizers can replace 23–52% of nitrogen fertilizers without causing a loss of yield (Rose et al., 2014). In this study, we found that a 30% or 40% reduction in chemical fertilizers combined with 6,000 or 9,000 kg ha^–1^ bio-organic fertilizer did not cause a significant loss of yield, and the yields of the T1 and T2 treatment groups were higher than that of the control groups with conventional chemical fertilization (Figures 4A,B). Thus, our results reveal the potential role of bio-organic fertilizers in reducing the chemical fertilizer load in soil. Similar results have also been reported in cabbages (Sarkar and Rakshit, 2021), cucumbers (Zhao et al., 2018), tomatoes (Ye et al., 2020b), peppers (Wu et al., 2015), and other crops, and the use of bio-organic fertilizers has also been shown to increase crop yield.

**FIGURE 5:**
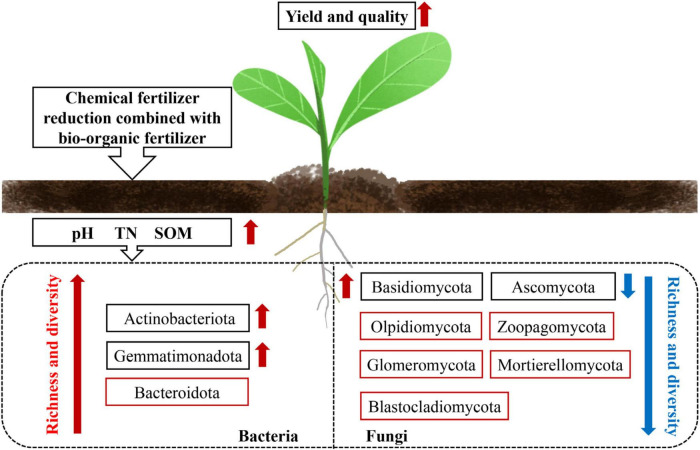
Potential mechanism of action of reduced chemical fertilizer combined with bio-organic fertilizer in regulating soil microecology to improve the yield and quality of lettuce. The red arrow indicates an increase, and the blue arrow indicates a decrease. The red box indicates bacterial or fungal phyla with significant differences between treatments.

Human diets containing vegetables with a high nitrate content can cause serious health hazards. Excessive application of mineral fertilizers—especially N fertilizers—is the main reason for high levels of nitrate accumulation in plant tissues (Wang et al., 2008). In the present study, the nitrate content of the T1–4 treatment groups was significantly lower (by 25.25–32.88%) than that of the CK group. This may be because the improvement in soil microorganisms reduces the nitrate content by increasing the activity of nitrate reductase, thus improving the safety and quality of vegetables (Chatterjee et al., 2012). Qi et al. (2021) found that the levels of soluble sugars and vitamin C increased in “yellow rose” cabbage treated with bio-organic fertilizer. In this study, we found that in lettuce, the levels of soluble sugars, soluble protein, and vitamin C increased to varying degrees in the T1–4 treatment groups (Figure 4). Similar results have also been reported in tomatoes (Brunetti et al., 2019) and cauliflowers (Yang et al., 2020a). Based on these findings, we speculate that the application of reduced chemical fertilizer combined with bio-organic fertilizer can promote the yield and quality of lettuce by improving soil chemical properties and microbial communities (Figure 5).

## Conclusion

In the present study, we revealed that the reduction of chemical fertilizer combined with bio-organic fertilizer prevented soil acidification and effectively improved the soil’s chemical properties. Treatments with reduced chemical fertilizer combined with bio-organic fertilizer increased the richness and diversity of the bacterial community and decreased those of the fungal community. The PCoA analysis results showed that the bacterial and fungal communities were separated between the treatment (reduced chemical fertilizer combined with bio-organic fertilizer) and CK groups. Different levels of reduced chemical fertilizers combined with bio-organic fertilizers also had an impact on the composition of the bacterial and fungal communities. The Kruskal–Wallis *H*-test showed that significant differences were more evident in the fungal phyla than in the bacterial phyla among the treatment groups, suggesting that the fungal community was more sensitive to reductions in chemical fertilizer combined with bio-organic fertilizer. The results of the RDA and Mantel test analysis indicated that the short-term impact of organic fertilizers on the soil microbial community was milder than that of chemical N fertilizer. Further, reduced chemical fertilizer combined with bio-organic fertilizer did not significantly affect the lettuce yield or increase it, and improved its safety and nutritional quality. Current agricultural practices present a contradiction between the rapid improvement of soil fertility and the reduction of pollution by chemical fertilizers. Against this background, the combined application of reduced chemical fertilizer and bio-organic fertilizer effectively improved soil fertility and microbial community structure and promoted the yield and quality of lettuce. This study provides a scientific basis for establishing an environmentally friendly fertilization technology and effectively promoting sustainable agriculture.

## Data Availability Statement

The datasets (SRP359012) presented in this study can be found in the NCBI Sequence Read Archive (https://identifiers.org/ncbi/insdc.sra:SRP359012).

## Author Contributions

NJ, JLy, and JY designed the study. NJ, LJ, SW, and JLi performed the work. NJ was involved with writing the manuscript. NJ, LJ, and FL analyzed the data. ZL, SL, YW, JLy, and JY revised the manuscript. All authors contributed to the article and approved the final version of this manuscript.

## Conflict of Interest

The authors declare that the research was conducted in the absence of any commercial or financial relationships that could be construed as a potential conflict of interest.

## Publisher’s Note

All claims expressed in this article are solely those of the authors and do not necessarily represent those of their affiliated organizations, or those of the publisher, the editors and the reviewers. Any product that may be evaluated in this article, or claim that may be made by its manufacturer, is not guaranteed or endorsed by the publisher.

## Footnotes

1
https://github.com/OpenGene/fastp

2
https://ccb.jhu.edu/software/FLASH/index.shtml

3
http://drive5.com/uparse/

4
http://sourceforge.net/projects/rdp-classifier/
